# Prevalence of Diabetes in Low‐ and Middle‐Income Countries' Incarcerated Populations: A Systematic Review and Meta‐Analysis

**DOI:** 10.1002/edm2.70374

**Published:** 2026-11-01

**Authors:** Saad Arsalan Wasti, Areesha Wasti, Alisha Ahmed, Beenish Sabir, Ariba Asif, Sardar Noman Qayyum, Hammad Naveed, Noor ul Ain Saleem, Muhammad Usman Iqbal, Hamayle Saeed, Dori Woldu, Shahad Abu Ahmad, Muhammad Nabeel Saddique

**Affiliations:** ^1^ King Edward Medical University Lahore Pakistan; ^2^ Continental Medical College Lahore Pakistan; ^3^ Jinnah Sindh Medical University Karachi Pakistan; ^4^ Rawalpindi Medical University Rawalpindi Pakistan; ^5^ Bacha Khan Medical College Mardan Pakistan; ^6^ FMH College of Medicine and Dentistry Lahore Pakistan; ^7^ Department of Internal Medicine St. Elizabeth Youngstown Hospital, Mercy Health Youngstown Ohio USA; ^8^ Butaro Level Two Teaching Hospital Burera Rwanda; ^9^ Al‐Quds University East Jerusalem Palestinian Territory, Occupied

**Keywords:** diabetes mellitus, LMIC, meta‐analysis, prevalence, prison health

## Abstract

**Background and Aims:**

Diabetes mellitus (DM) is a major global health concern, particularly in resource‐constrained settings. Incarcerated populations in low‐ and middle‐income countries (LMICs) face increased health risks and limited care access, yet remain understudied. No prior systematic review has synthesized prevalence data for this group. This review aims to estimate diabetes prevalence among incarcerated individuals in LMICs.

**Methods:**

We conducted a systematic review and meta‐analysis per PRISMA and GATHER guidelines. PubMed, Scopus, Embase, Web of Science and regional databases were searched till May 2025. Random‐effects models with Freeman‐Tukey double arcsine transformation were applied in R (version 4.5.1). Publication bias was assessed using DOI plots. Study quality was evaluated with the Joanna Briggs Institute tool, and certainty of evidence was appraised using an adapted GRADE approach.

**Results:**

Twenty‐three studies (107,473 participants) were included. Mean participant ages varied from 29 ± 9.6 to 40.5 ± 9.8 years. The tentative pooled estimate of diabetes prevalence was 3.7% (95% CI: [2.5%–5.1%], Prediction Interval [0%–12.2%]), with high heterogeneity (*I* ^2^ = 95.4%). The pooled prevalence estimate was highest in Asia (5.2%), particularly Pakistan (9.8%), and lowest in South America (2.3%). The DOI plot revealed major asymmetry, indicating a high possibility of publication bias. Overall certainty of evidence was rated as very low for diabetes prevalence due to risk of bias and publication bias. Interpretation of the pooled estimate is limited by substantial heterogeneity and limitations in the primary studies, including differences in diabetes ascertainment methods.

**Conclusion:**

Diabetes is an important health concern among incarcerated populations in LMICs. Differences in diabetes ascertainment methods, participant characteristics and correctional healthcare systems may have potentially contributed to the variability of the pooled estimates. A hypothesis is that there may also be potentially limited detection or underdiagnosis which possibly could have contributed to the observed prevalence estimates.

## Graphical abstract

Diabetes prevalence varied considerably across incarcerated populations in low‐ and middle‐income countries, with high heterogeneity. Differences in diabetes ascertainment highlight the need for standardized diagnostic criteria and consistent reporting to improve the comparability of future estimates.

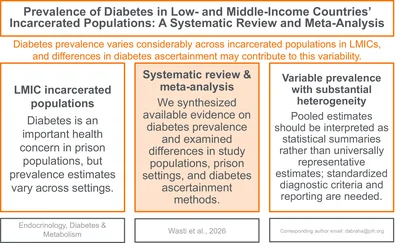

## 1 Introduction

Diabetes mellitus (DM) comprises metabolic disorders characterized by chronic hyperglycemia resulting from defects in insulin secretion and/or action [1]. It may arise from beta‐cell destruction causing insulin deficiency or increased insulin resistance with relative insulin deficiency, leading to hyperglycemia [2].

According to the 2024 update of American Diabetes Association (ADA), DM is diagnosed if any of the following are present: A1C level ≥ 6.5% (48 mmol/mol), fasting blood glucose FPG of ≥ 126 mg/dL (7.0 mmol/L) and 2‐h glucose (2‐h PG) level during a 75‐g oral glucose tolerance test (OGTT) of ≥ 200 mg/dL (11.1 mmol/L) and in an individual with classic symptoms of diabetes (such as unexplained weight loss, polydipsia, or polyuria) or hyperglycemic crisis, a random plasma glucose level of ≥ 200 mg/dL [3].

In 2022, it was estimated that about 828 million adults were living with diabetes globally, reflecting an increase of 630 million compared to 1990 [4]. The increasing trend was found to be significantly influenced by low‐ and middle‐income countries (LMIC). According to Sun et al., the global prevalence of DM is expected to rise to 12.2% by 2045, and middle‐income countries are likely to have the greatest relative increase in prevalence between 2021 and 2045 [5].

Diabetes treatment coverage has not increased sufficiently in most LMICs in comparison to the rise in the disease prevalence, resulting in continued increase in the burden of untreated diabetes and its complications [4]. Lower socioeconomic status is associated with higher mortality and morbidity in DM patients [6]. Chronic complications responsible for significant mortality and morbidity include retinopathy, nephropathy, neuropathy, cardiovascular disease, stroke, peripheral artery disease (PAD) and other complications such as decreased resistance to infections, dental diseases and birth‐related complications of gestational diabetes [7, 8].

About 11.5 million people are incarcerated in detention centres worldwide, and the figures continue to rise [9]. Incarcerated individuals may face greater challenges than the general population despite their undisputed right to healthcare. This could possibly include factors such as their low socioeconomic standing and social marginalization. Many chronic illnesses, such as obesity, hypertension and diabetes and transmitted infections, such as hepatitis B and C, HIV and tuberculosis, are more prevalent in the incarcerated population [10, 11]. Furthermore, poor health‐related quality of life (HR‐QOL) among incarcerated individuals has also been found to be associated with the substandard living conditions of the detention centre [12, 13].

Although the prevalence of DM among the inmates was found to be similar to the national prevalence [14, 15, 16, 17], *Simeni‐Njonnoue* et al. reported increased prevalence of DM among inmates attributed to poor nutritional conditions and sedentary lifestyles. Other identified risk factors include smoking, hypertension, obesity and detention‐related variables such as sentence for major or minor crime and reason for incarceration [18].

Insufficient medical personnel and physical health promotion initiatives, substandard equipment, inconsistent screening processes, unavailability of medications and nutritional counselling, and exercise noncompliance in detention centres contribute to poor standards of healthcare for diabetic inmates and complicate diabetes management [19, 20].

To date, no pooled analysis has assessed the prevalence of diabetes among the incarcerated individuals of LMIC. Additionally, certain regions and countries are severely underrepresented in international literature and are sometimes omitted from evidence synthesis. In order to raise awareness, advocate for these neglected populations, inform healthcare policies and allocate appropriate funding to manage the disease burden, an accurate estimate of the prevalence of diabetes in the incarcerated population in LMIC is necessary.

## 2 Methods

This systematic review and meta‐analysis was conducted in line with the protocol registered on PROSPERO (registration number: CRD420251082497) [21] and reported according to the Preferred Reporting Items for Systematic Reviews and Meta‐Analyses (PRISMA) 2020 guidelines [22], and the Guidelines for Accurate and Transparent Health Estimates Reporting (GATHER) reporting standards [23]. The completed PRISMA and GATHER checklists are available in the supplementary file (Tables S1 and S2). Ethics approval was not required for this review, as we obtained all data from publicly available sources.

### 2.1 Information Sources and Literature Search

Two authors independently searched four international databases (PubMed, Scopus, Embase and Web of Science) to retrieve all relevant records from the inception of respective databases to May 22, 2025. The search strings consisted of two search terms: (i) Diabetes; and (ii) Correctional Facilities. Controlled vocabularies (e.g., MeSH, Emtree) and keywords (e.g., ‘diabetes mellitus’, ‘jail’, ‘correctional facilities’, ‘prison’) were combined using BOOLEAN operators, truncations and database‐specific adaptations. Searches were performed without any language, design, year, or country restrictions, based on pilot searches showing that such filters excluded relevant studies. With regard to other sources, regional article indexes [Latin American and Caribbean Health Sciences Literature (LILACS), African Journals Online (AJOL), PakMediNet, Western Pacific Region Index Medicus (WPRIM), China National Knowledge Infrastructure (CNKI), Scientific Information Database (SID)] were also searched independently by two authors (S.A.W. and A.W.). Websites, including Global Index Medicus, were also searched to identify potentially relevant studies not indexed in the major databases. Citation chaining (forward and backward searching) was also performed for all relevant articles to find additional studies. All searches were validated by a third author. The line‐by‐line search strategy for each source has been provided in the supplementary file (Tables S3 and S4).

### 2.2 Study Selection Process

All retrieved records were uploaded to Rayyan.ai [24] for screening. Records were first deduplicated, and then screened independently by two authors against titles and abstracts. Disagreements were resolved through mutual discussion or a third reviewer when necessary. The full texts of the records considered potentially eligible for inclusion were screened independently by two reviewers. Conflicts were resolved through discussion or a third author if needed. Articles published in languages other than English were included if an official/published English translation was available through the publisher or could be identified via indexing platforms, DOI links, or online repositories. The study was excluded if translation was not available through indexed sources. Screening decisions were made based on predefined inclusion and exclusion criteria.

### 2.3 Eligibility Criteria

Inclusion criteria were studies that: (1) Reported the prevalence of diabetes mellitus among incarcerated people, or provided sufficient information to calculate its prevalence; (2) Included an incarcerated population sufficiently representative of the general incarcerated population; (3) Took place in low‐ and middle‐income countries (LMICs) only; and (4) Were published in the English language, or had a published/official English translation.

Exclusion criteria were studies that: (1) Reported the prevalence of diabetes mellitus among non‐incarcerated population, unless the diabetes prevalence for incarcerated population can be isolated; (2) Only reported the prevalence of diseases other than diabetes mellitus (such as diabetes insipidus, hypertension, tuberculosis); (3) Included a non‐representative incarcerated sample (e.g., pregnant‐only incarcerated women, tuberculosis‐only incarcerated people, people testing positive for Coronavirus disease 2019 [COVID‐19] etc.); (4) Took place in high‐income countries; and (5) Were not published in English language, or had no published/official English translation.

Where the prison population was divided according to tuberculosis status but the entire study population was the incarcerated population, diabetes cases from the eligible groups were considered together to derive the prevalence estimate for the sampled prison population.

### 2.4 Outcomes Assessment

The main outcome of this systematic review and meta‐analysis was to estimate the pooled prevalence of diabetes across all low‐ and middle‐income countries (LMICs). Countries were classified according to the World Bank country income classification for fiscal year 2024–2025 [25]. This predefined classification was applied consistently to the countries represented in the included studies, rather than assigning income status according to the historical publication date of each study, or according to the year of the start of the data collection of the original study, or according to the year of the end of the data collection process of the original study. Subgroup analyses were planned for WBIB status, country, continent and type of correctional facility to evaluate differences in prevalence estimates across the aforementioned potential sources of heterogeneity. Secondary outcomes included evaluating the prevalence of individual subtypes of diabetes and prediabetes.

### 2.5 Data Extraction

One reviewer prepared a preliminary data extraction sheet on Google Sheets and another reviewer further refined it. The sheet was then pilot‐tested to ensure its suitability for the review. Feedback from the pilot testing was utilized to further improve the sheet. Two reviewers then independently extracted data for all studies on separate sheets.

Data extracted included key study characteristics (author, year, total sample size, country, world bank income bracket, continent, study design, study centres, facility centre, methods of recording diabetes diagnosis, methods of diagnosing diabetes and types of diabetes studied), key patient demographics (age, biologic sex, body mass index and height). Data extracted for outcomes were the overall prevalence of diabetes, individual prevalences of the subtypes of diabetes, and prediabetes. Where continuous participant characteristics were reported in different formats, the Meta‐converter tool was used, where required, to convert these data into a common format for presentation in the baseline characteristics [26]. The tool was not used to derive or transform the prevalence estimates included in the meta‐analysis. Once the extraction was complete, the discrepancies were resolved through discussion.

### 2.6 Quality Assessment

Two reviewers independently appraised the quality of the included studies. A third reviewer acted as an adjudicator for unresolved discrepancies. The JBI appraisal tool was used for quality assessment [27]. The appraisal tool comprises nine criteria to assess study quality. These included the appropriateness of the sample frame, participant recruitment methods, sample size adequacy, detailed description of subjects and settings, data coverage of the sample, validity of condition identification, consistency in measurement, appropriateness of statistical analysis and adequacy and management of response rates. Representativeness was judged from the information reported in each study and was not inferred solely from sample size.

### 2.7 Statistical Analysis

All statistical analyses were performed independently by two reviewers using R, version 4.5.1 [28]. Prior to conducting analyses, specialized packages in R were loaded, including the ‘meta’, ‘metafor’, ‘metasens’, ‘readxl’, ‘openxlsx’, ‘dmetar’ and ‘ggplot2’ packages. A random‐effects model was used to pool prevalences, with 95% confidence intervals (CI) and prediction intervals (PI). The Freeman‐Tukey double arcsine transformation was used to stabilize variances. Because prevalence proportions may be skewed, particularly for estimates close to 0 or 1, individual study proportions were transformed using the Freeman Tukey double‐arcsine transformation. Transformed estimates were pooled using inverse‐variance weighting under a random‐effects model. Between‐study variance (*τ* ^2^) was estimated using restricted maximum likelihood (REML). A post hoc sensitivity analysis, a generalized linear mixed‐effects model (GLMM), was also conducted and compared with the primary analysis.

The *I* ^2^, *τ* ^2^ and *χ* ^2^ tests with degrees of freedom and *p*‐value were used to evaluate heterogeneity. Heterogeneity was categorized using the following *I* ^2^ intervals: < 25% as low, ≥ 25% and < 50% as moderate and ≥ 50% as high. Select, preplanned subgroup analyses were conducted in line with guideline recommendations. Meta‐regressions were also conducted using the covariates sample size and publication year. Leave‐one‐out sensitivity analyses and *Baujat* plots were also performed to further explore heterogeneity and influence of outliers. The DOI plot with LFK index was used to assess publication bias. Any discrepancies during analysis were resolved through mutual discussion.

### 2.8 Certainty of Evidence

There currently exists no formal guidance regarding implementing a GRADE‐based approach to systematic reviews and meta‐analyses of prevalence. However, given the need for a formal assessment of study quality and certainty of evidence for this review, due to global health implications, the evidence body was appraised using an adapted GRADE approach. Guidelines were taken from general GRADE guideline papers, the GRADE handbook, the new GRADE book and published prevalence systematic reviews and meta‐analyses. A detailed breakdown of the adapted‐GRADE approach is provided in the supplementary file (Table S5). The GRADEpro software was used to organize data and judgements in the form of a Summary of Findings (SoF) table.

## 3 Results

### 3.1 Study Selection Process

A total of 2042 records were identified through database searching (*n* = 1974) and other sources (*n* = 68). From the articles retrieved from database search, after removal of duplicates (*n* = 1017), 957 records from the database search were screened by title and abstract, of which 890 were excluded for not meeting inclusion criteria. Full‐text assessment was conducted for 67 articles, and 17 were included. An additional 6 studies were identified through other sources. Ultimately, 23 studies were included in this review [29, 30, 31, 32, 33, 34, 35, 36, 37, 38, 39, 40, 41, 42, 43, 44, 45, 46, 47, 48, 49, 50, 51]. The study selection process is illustrated in the Preferred Reporting Items for Systematic Reviews and Meta‐Analyses (PRISMA) flow diagram (Figure 1).

**FIGURE 1:**
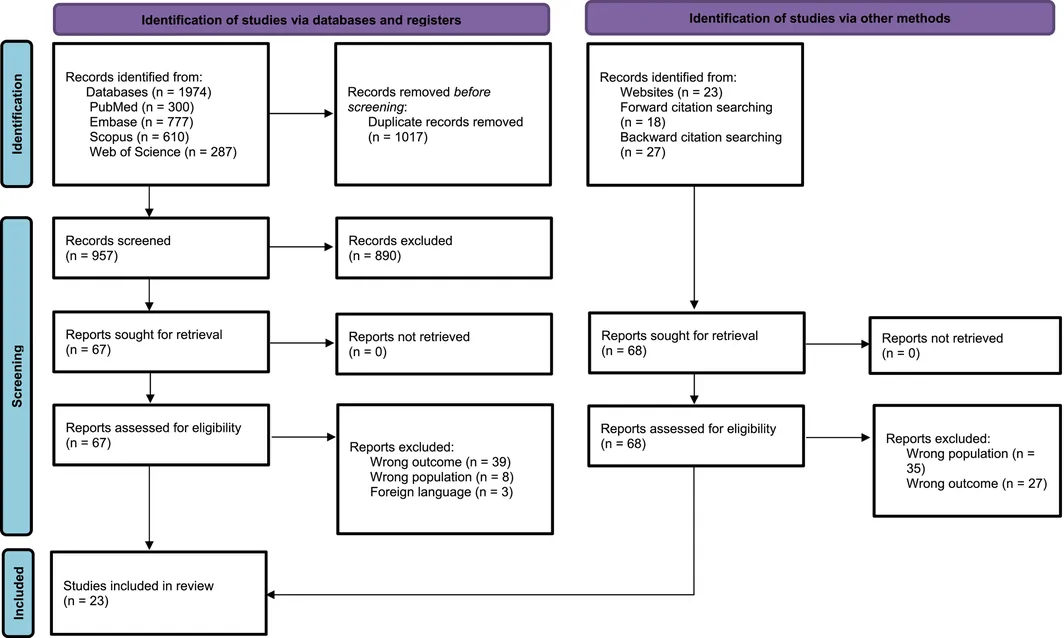
PRISMA flowchart, showing results of the study selection process.

### 3.2 Characteristics of Included Studies

Table 1 lists the key characteristics of the included studies. The 23 included studies represented 17 countries across 5 continents. The studies were primarily from Asia (*n* = 10) and South America (*n* = 6), followed by Africa (*n* = 5), North America (*n* = 1) and Europe (*n* = 1). Most studies were conducted in upper‐middle‐income (*n* = 11) or lower‐middle‐income (*n* = 9) countries. A smaller number were from low‐income countries (*n* = 3).

**TABLE 1:** Table showing key study characteristics and patient demographics.

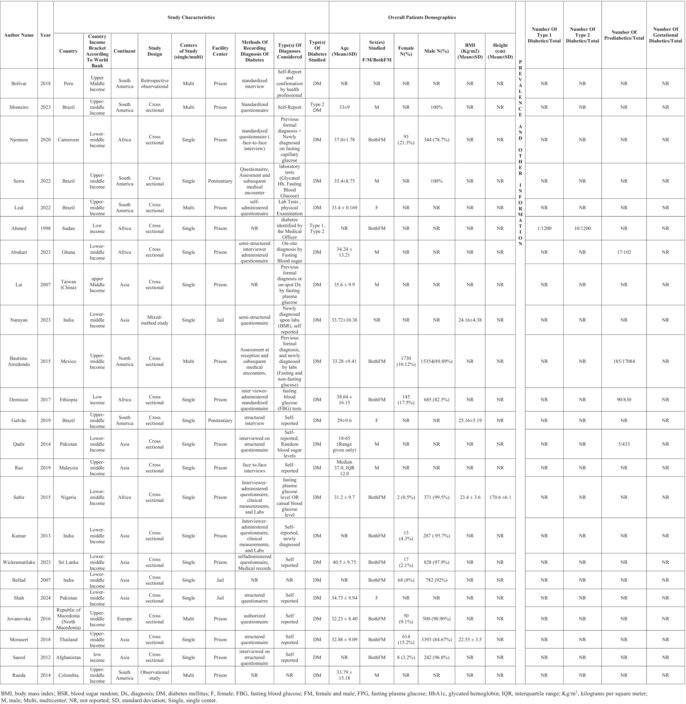

Diagnostic approaches for diabetes mellitus (DM) varied, including self‐reported diagnoses, medical records and clinical assessments. Most studies reported diabetes prevalence without specifying type; a minority reported subtypes or prediabetes (Table 1).

### 3.3 Assessment of Study Quality

Study quality was appraised using the Joanna Briggs Institute (JBI) Critical Appraisal Checklists. Overall, study quality was variable (ranging from low to high). Common methodologic limitations included inadequate sample size, usage of convenience sampling and reliance on self‐reported diagnoses. Detailed assessments are presented in Table S6.

### 3.4 Outcomes

Twenty‐three studies comprising a total of 107,473 participants were included in the pooled prevalence analysis. The pooled prevalence of diabetes mellitus in the incarcerated population was 3.7% (95% CI: [2.5% to 5.1%], *I* ^2^ = 95.4%, *p* < 0.0001) (Figure 2). There was high inter‐study heterogeneity.

**FIGURE 2:**
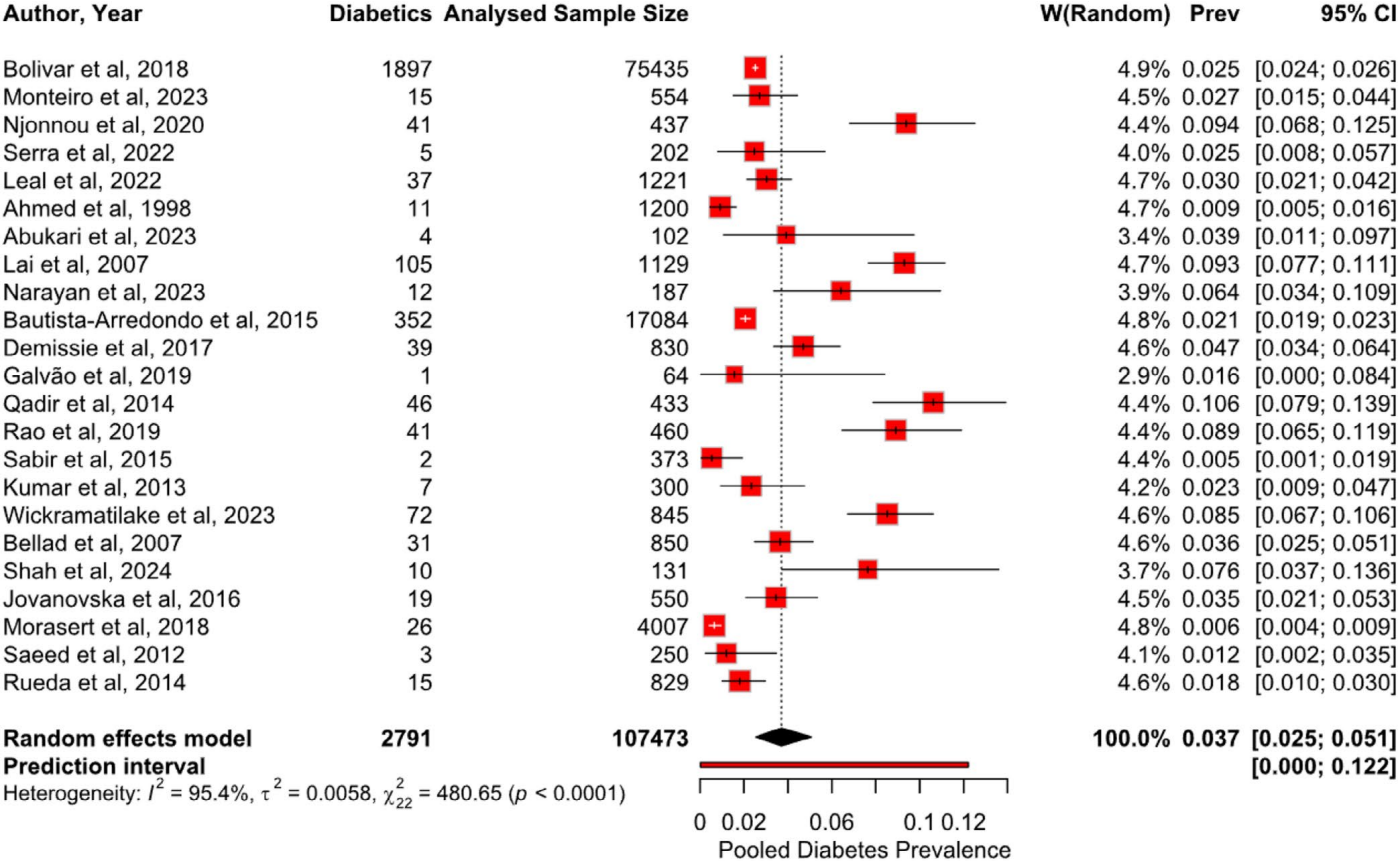
Forest plot showing pooled prevalence of diabetes mellitus in prisoners.

Subgroup analysis by World Bank Income Bracket (WBIB) showed a pooled diabetes prevalence of 3.1% in upper‐middle‐income, 5.3% in lower‐middle‐income, and 2.0% in low‐income countries, with no statistically significant difference between groups (*p* = 0.1249) (Figure 3). Subgrouping by continent, the pooled prevalence was 5.2% in Asia, 3.2% in Africa, 2.3% in South America, 2.1% in North America and 3.5% in Europe. Differences between continents were statistically significant (*p* < 0.0001) (Figure 4). When analysed by country, prevalence estimates ranged from 0.5% in Nigeria to 9.8% in Pakistan, with statistically significant variation across countries (*p* < 0.0001) (Figure 5). By facility type, the pooled prevalence was 3.6% in prisons, 2.1% in penitentiaries and 5.3% in gaols. These differences were not statistically significant (*p* = 0.2117) (Figure S1).

**FIGURE 3:**
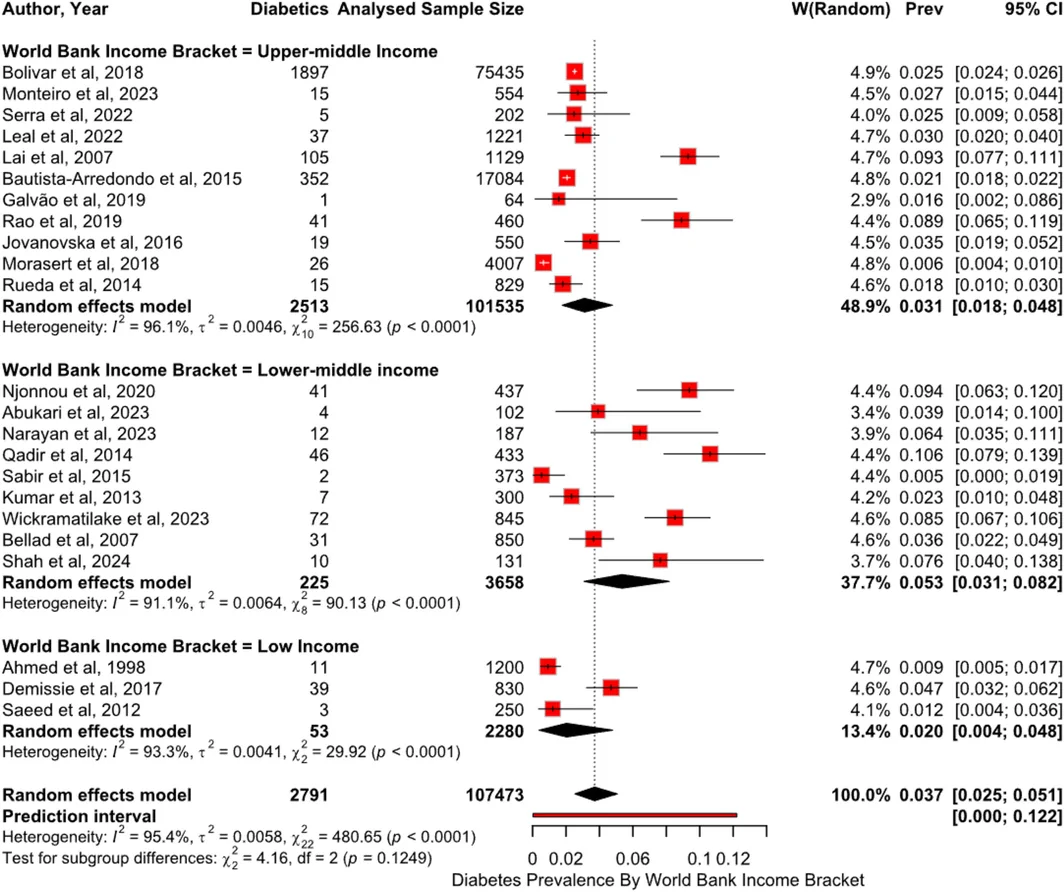
Forest plot showing pooled prevalence of diabetes mellitus in prisoners, subgrouped by World Bank Income Bracket (WBIB).

**FIGURE 4:**
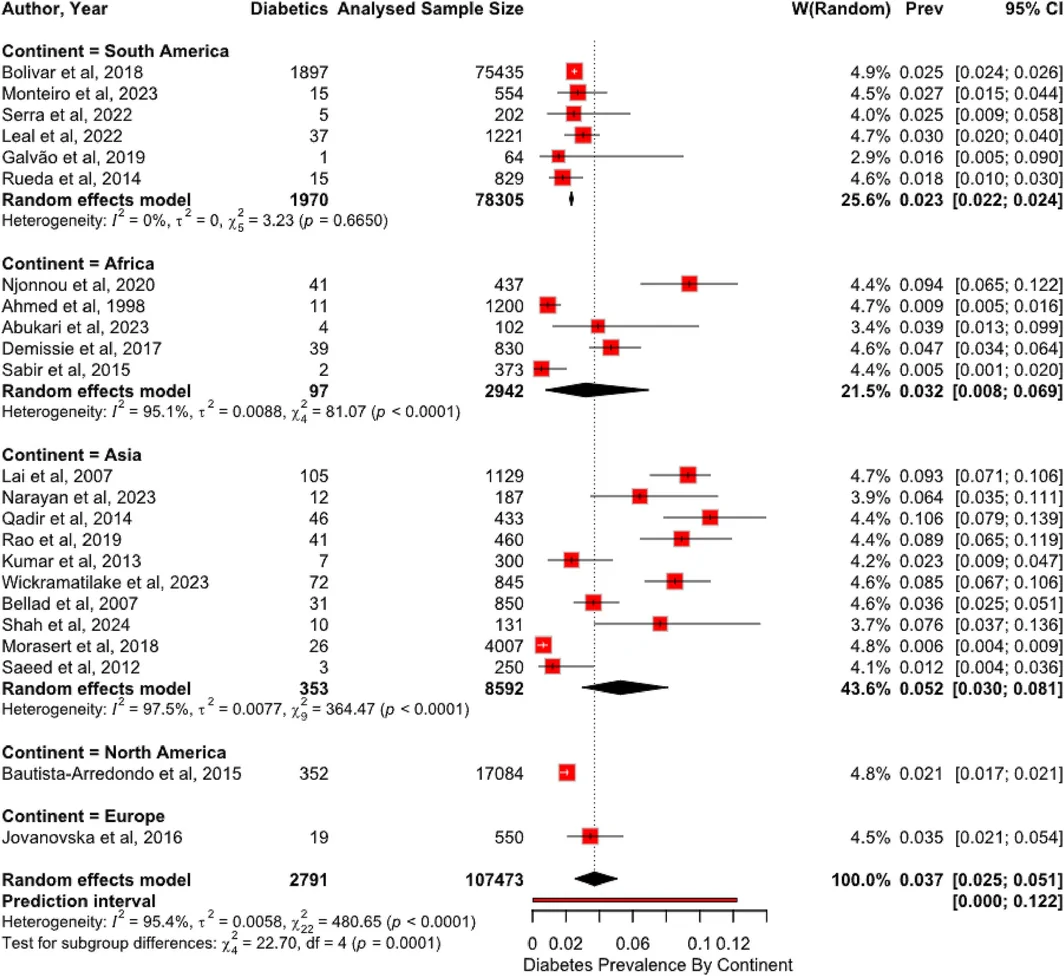
Forest plot showing pooled prevalence of diabetes mellitus in prisoners, subgrouped by continent.

**FIGURE 5:**
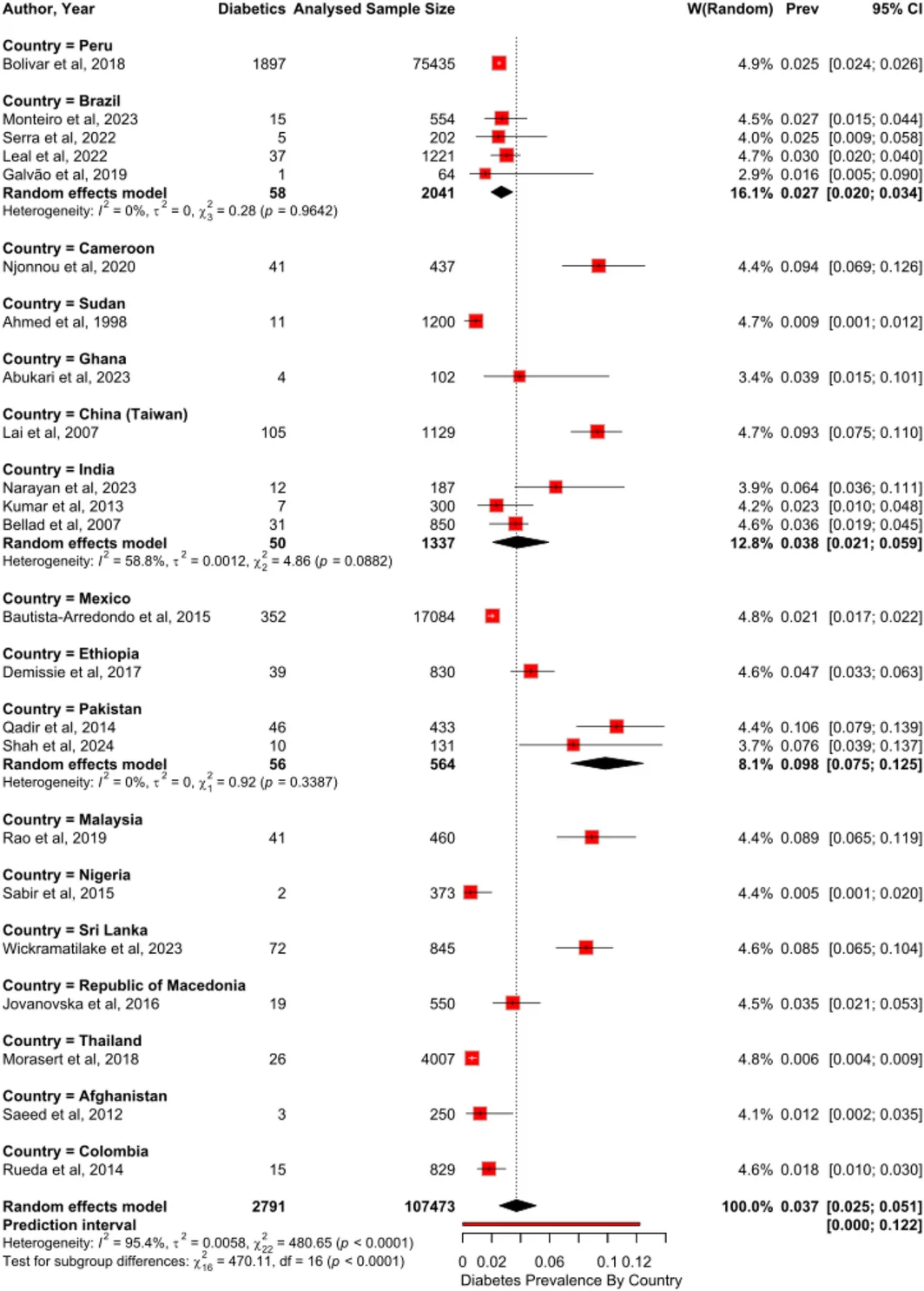
Forest plot showing pooled prevalence of diabetes mellitus in prisoners, subgrouped by country.

Data on diabetes subtypes were limited. One study conducted in Sudan reported prevalence estimates of 0.08% (1/1200) for Type 1 diabetes and 0.83% (10/1200) for Type 2 diabetes. Four studies reported prediabetes, with estimates ranging from 1.08% to 16.7%. No studies reported on gestational diabetes. Due to insufficient reporting, pooled analyses for subtypes and prediabetes were not conducted (Table 1).

### 3.5 Exploration of Heterogeneity

#### 3.5.1 Sensitivity Analyses

Sensitivity analyses were conducted through leave‐one‐out method to assess the influence of individual studies on the overall pooled diabetes prevalence. Omitting each study sequentially did not substantially alter the summary estimate, which remained stable between 3.5% and 3.9%, with overlapping confidence intervals across all iterations. The overall prevalence estimate ranged from 3.5% (95% CI [2.4% to 4.8%]) to 3.9% (95% CI [2.7% to 5.3%]), with *I* ^2^ consistently > 94%, indicating persistent high heterogeneity regardless of the study excluded. No single study substantially influenced the summary estimate (Figure S2). Additionally, as a post hoc sensitivity analysis, a generalized linear mixed‐effects model (GLMM) yielded a pooled diabetes prevalence of 3.2% (95% CI 2.3%–4.6%), compared with 3.7% (95% CI 2.5%–5.1%) in the primary Freeman‐Tukey analysis. Heterogeneity also remained high (*I* ^2^ = 96.4%), indicating that the overall findings were broadly consistent across analytical approaches.

#### 3.5.2 Baujat Plot

The *Baujat* plot indicated that no individual study had a disproportionate effect on either heterogeneity or overall effect size. Contributions to the *Q* statistic were all < 3 and influence < 0.15 (Figure S3).

#### 3.5.3 Meta‐Regression

Meta‐regression was also performed to explore whether any covariates accounted for variability in the prevalence estimates. The findings were visualized using bubble plots.

#### 3.5.4 Covariate: Year of Publication

Meta‐regression showed no significant association between year of publication and diabetes prevalence (*β* = 0.0033, 95% CI [−0.0019 to 0.0085], *p* = 0.2139). The model explained only 2.1% of heterogeneity (*R* ^2^ = 2.10%), with residual heterogeneity remaining high (*τ* ^2^ = 0.0057; *I* ^2^ = 97.96%; *H* ^2^ = 49.08) (Figure S4).

#### 3.5.5 Covariate: Sample Size

Sample size was also not a significant moderator (*β* = −0.0000, 95% CI [−0.0000 to 0.0000], *p* = 0.4660, *R* ^2^ = 0.00%). Residual heterogeneity remained substantial (*τ* ^2^ = 0.0059, *I* ^2^ = 96.5%, *H* ^2^ = 28.53) (Figure S5). A sensitivity regression using log‐transformed sample size also yielded non‐significant results.

### 3.6 Publication Bias

Publication bias was assessed using a DOI‐plot with the Luis Furuya‐Kanamori (LFK) index (Figure 6). The DOI‐plot revealed marked asymmetry, with an LFK index of 3.58, indicating major asymmetry. According to accepted thresholds, an LFK index > ±2 is considered to reflect major asymmetry [52], suggesting potential publication bias or small‐study effects.

**FIGURE 6:**
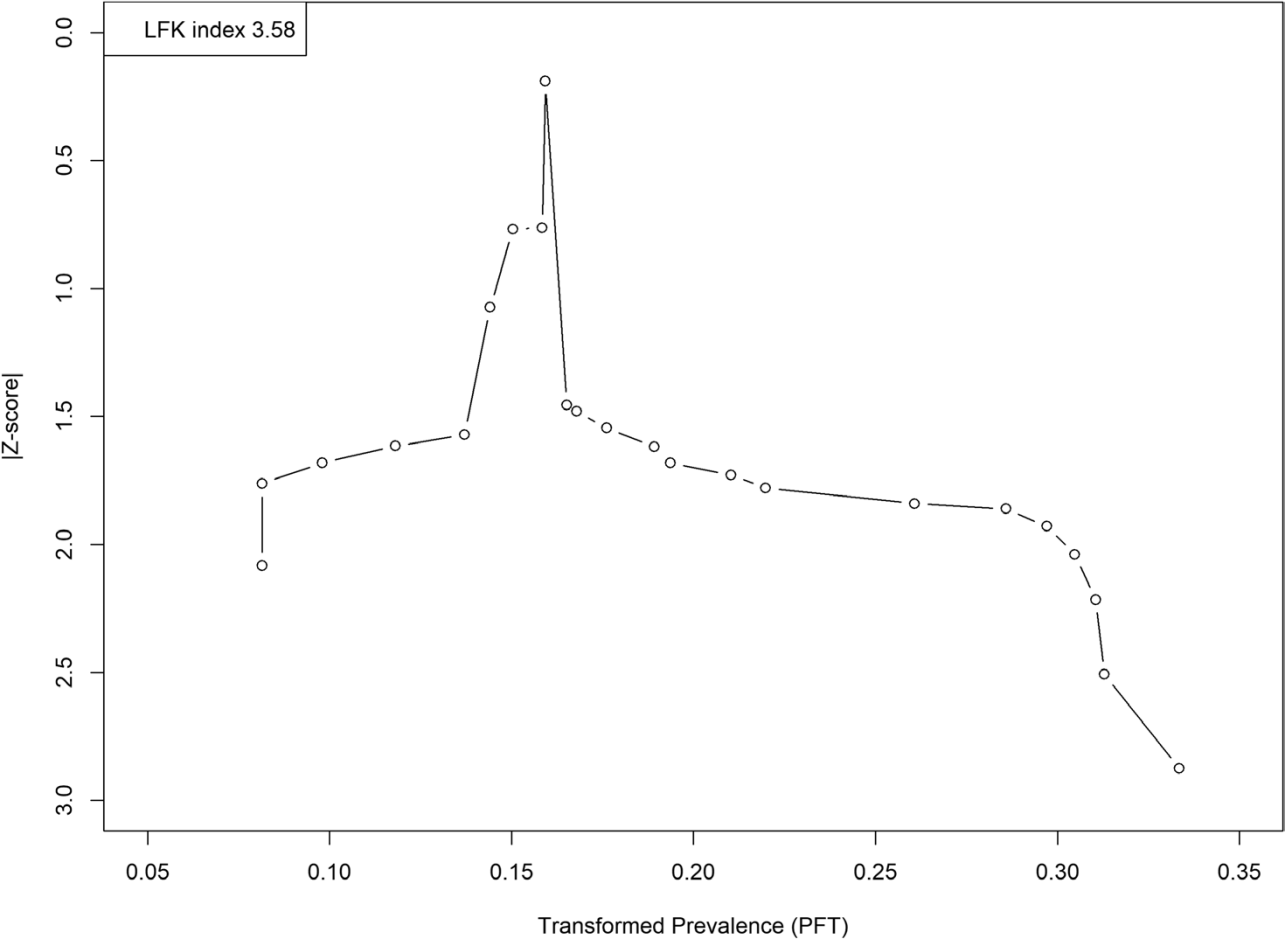
DOI plot with Luis Furuya‐Kanamori (LFK) Index, showing marked asymmetry.

### 3.7 GRADE Certainty of Evidence (Adapted GRADE Approach)

An adapted GRADE approach was used to appraise the certainty of evidence, with the results presented in the form of a Summary of Findings (SoF) table (Table 2). After applying downgrades for risk of bias (due to reliance on self‐report in a considerable number of studies) and publication bias (as indicated by LFK index > 3.0), the overall certainty of evidence for pooled diabetes prevalence in incarcerated individuals was rated as very low. The certainty of evidence for the prevalence of prediabetes was rated as very low for similar limitations (Table 2). However, because the adapted GRADE approach has not been formally validated for prevalence studies, these certainty of evidence ratings should be considered exploratory rather than definitive.

**TABLE 2:** Summary‐of‐findings (SoF) table, showing evidence appraisal using an adapted‐GRADE approach.

Prevalence of diabetes in prisoners of low‐ and middle‐income countries (LMICs)			
Outcome no of participants (Studies)	Prevalence (%)	Certainty of evidence	What it means
Pooled diabetes prevalence no of participants: 107,473 (23 Studies)	Pooled diabetes prevalence in prisoners was 3.7% (95% CI [2.5% to 5.1%], PI [0.0% to 12.2%]).	⨁◯◯◯ Very low^a^, ^b^	Although the large pooled sample size yielded an estimated prevalence of 3.7%, limitations in study quality and high possibility of publication bias suggest that the true prevalence may differ substantially from this estimate.
Prevalence of prediabetes no of participants: 32,109 (4 Studies)	Prevalence estimates in individual studies ranged from 1.08% to 16.7%.	⨁◯◯◯ Very low^c^, ^d^	The evidence is very uncertain about the prevalence of prediabetes in the prisoners of low‐ and middle‐income countries (LMICs).

*Note:* GRADE Working Group grades of evidence. High certainty: we are very confident that the true effect lies close to that of the estimate of the effect. Moderate certainty: we are moderately confident in the effect estimate: the true effect is likely to be close to the estimate of the effect, but there is a possibility that it is substantially different. Low certainty: our confidence in the effect estimate is limited: the true effect may be substantially different from the estimate of the effect. Very low certainty: we have very little confidence in the effect estimate: the true effect is likely to be substantially different from the estimate of effect.

Abbreviations: CI: confidence interval; LMICs: low‐ and middle‐income countries; PI: prediction interval.

^a^ Downgraded twice for risk of bias: a significant number of studies had limitations, including use of self‐reported method of diagnosis.

^b^ Downgraded for publication bias: major asymmetry was seen in the DOI plot (LFK index 3.58).

^c^ Downgraded for risk of bias: studies assessing prediabetes did not show explicit evidence of repeat testing on multiple occasions, which limits the reliability of the calculated prevalence estimates.

^d^ Downgraded twice for imprecision: first for sparse data (only 4 studies, spanning select countries, which restrict generalizability and firm conclusions); then for high inconsistency (extremely wide discrepancies in reported prevalence estimates between studies, which greatly limit reliable interpretations).

## 4 Discussion

This systematic review and meta‐analysis assesses the prevalence of diabetes within the incarcerated population of LMIC, a demographic that is frequently underrepresented in public health research and is considered particularly vulnerable. Our pooled analysis estimated an overall prevalence of diabetes in this population of 3.7%, less than the 7.1% found in previous meta‐analyses, whose pooled global estimates were significantly biassed toward high‐income nations, especially the US [53, 54, 55]. This discrepancy is likely explained by the differences attributed to the variation in geographical distribution between the sources included in our study, as prior analyses were heavily weighted toward high‐income countries, particularly the United States, thereby skewing the pooled analysis upward [53, 54, 55]. In contrast, our analysis focuses specifically on studies from LMIC settings across Asia, South America and Africa [17, 18].

Diabetes prevalence for the general adult population is estimated at 10.5% globally, which is expected to rise to 12.2% by 2045 [56]. Additionally, Munday et al. documented a diabetes prevalence of approximately 14% among older incarcerated individuals, which aligns closely with the prevalence observed in the general community [57]. Diabetes risk and outcomes are influenced by several factors, including socioeconomic position, gender distribution, demographic structure and access to healthcare, all of which are significantly different in the gaoled population than in the general community. Cardiometabolic risk is further increased by lifestyle variables such as a poor diet, little exercise, psychological stress and high smoking rates. Despite frequent updates on the prevalence of diabetes in the general community, research and surveillance continue to overlook gaoled individuals, especially in LMICs. The significant heterogeneity we found in our analysis (I^2 = 95.4%) most likely reflects diversity in research design, diagnostic criteria, healthcare capability and regional epidemiology.

Our study estimated a prevalence of diabetes in the incarcerated population of low and middle‐income countries at 3.7%. This is lower than the prevalence found in the general populations by some reviews. For instance, Hwang et al. reported a prevalence of diabetes in the rural population of LMICs as approximately 5.6% [58]. It cannot be clearly said, however, whether this is a true difference or involves other factors such as inadequate diagnostic methods and underreporting. Delays in diagnosis and inadequate treatment in LMIC correctional facilities are caused by structural obstacles such as a lack of qualified medical staff, a lack of diagnostic tools, uneven access to necessary medications and disjointed referral systems. The risk of Type 2 diabetes is further increased in incarcerated populations by socioeconomic deprivation, which includes poorer educational attainment, income insecurity and precarious work [59].

Subgroup analysis by continents showed a significant prevalence of diabetes at 5.2% among the incarcerated population in Asia. Similarly, the general population in South Asia has experienced an elevated risk of diabetes over the past decades. Our analysis revealed a significant difference in diabetes prevalence among different countries, with Pakistan showing a particularly higher prevalence of about 9.8%, aligning with the prevalence rate in the general population [60].

Factors influencing diabetes prevalence may be classified as pre‐incarceration, prison‐related and detection‐related factors. These may potentially affect both the underlying occurrence of diabetes and its detection through differences in screening, healthcare access and continuity of care. Thus, observed prevalence could reflect both disease burden and ascertainment. There are several reports of high blood pressure, smoking, physical inactivity and high intakes of sodium and refined carbohydrates in prison environments, all of which can raise the risk of cardiovascular disease [57, 61, 62, 63]. However, these results were drawn from studies conducted in high‐income countries with a paucity of data from LMIC incarcerated populations, reinforcing the need for context‐specific research.

Beyond individual risk factors, structural and policy determinants may contribute to shaping diabetes burden in correctional facilities. Prison health services in many LMICs continue to be administratively separate from national health systems, despite the UN Mandela Rules requiring equivalence of care. This may contribute to fragmented services, poor ongoing medical condition management and poor continuity of care following release. The World Health Organization advocates utilizing current HIV and TB programs to incorporate diabetes and hypertension diagnosis and has designated prisons as priority sites for integrated non‐communicable disease screening. Strengthening prison health systems may also be useful in achieving the Sustainable Development Goals (SDGs), particularly those related to health equity, universal health coverage and reducing inequalities, as prisons represent a key population that is often left behind in national health planning [64]. Since the majority of incarcerated individuals eventually return to society with unmanaged diabetes, failing to diagnose and treat the condition in correctional facilities may have consequences that go beyond incarceration. This includes an increased risk of complications, healthcare utilization and early mortality. Therefore, addressing diabetes in correctional facilities should be presented as a strategic public health investment, in addition to being a human rights requirement. Beyond estimating prevalence, future studies may consider focusing on gender‐sensitive analyses, standardized screening, post‐release care continuity and implementation tactics specific to LMIC correctional health systems.

Despite our study's smaller sample size compared to the previous study, we included a much wider global perspective of diabetes prevalence. This offers a broader estimate of diabetes prevalence in LMIC. Previous reviews relied predominantly on studies using large datasets from the US, which may not accurately reflect the global risk of diabetes among incarcerated individuals. Our analysis revealed substantial publication bias and very low certainty attributed to the included studies reporting non‐standardized or self‐reported diagnoses. However, this affects the generalizability of our findings. Furthermore, there were notable gaps in study‐level data and a lack of patient‐level data, which hindered us from pooling results based on the clinical type of diabetes. We only included studies from English‐language literature, which may have resulted in the exclusion of relevant data from non‐English literature.

### 4.1 Strengths

This review has several important methodological and contextual strengths. To our knowledge, this is the first systematic review and meta‐analysis to specifically quantify the prevalence of diabetes mellitus (DM) among incarcerated populations in low‐ and middle‐income countries (LMICs). By focusing exclusively on LMICs, this study addresses a critical evidence gap, as prior syntheses have been heavily weighted toward high‐income countries, particularly the United States, thereby limiting global applicability. Usage of prediction intervals and an adapted‐GRADE appraisal enhances the clinical applicability of our review. The review also captured studies from underrepresented countries and regions, providing a more comprehensive picture of the available LMIC evidence base.

### 4.2 Limitations

Several limitations should be considered when interpreting these findings. First, there was very high statistical heterogeneity, reflecting wide variability in reported prevalence across countries and settings. Although heterogeneity is expected in global prevalence syntheses, particularly across diverse correctional facility systems, it limits the interpretability of a single pooled estimate. The wide prediction interval (0% to 12.2%) suggests that future studies on incarcerated individuals in LMICs may find widely varying prevalence estimates, and the summary estimate should therefore not be interpreted as definitive or uniformly applicable across LMICs. Second, the diagnostic methods used to identify diabetes varied considerably. Many studies relied on self‐reported diagnoses or medical record abstraction rather than standardized biochemical testing (e.g., HbA1c or fasting plasma glucose). In settings with limited routine screening, this approach may potentially underestimate the true prevalence, particularly of undiagnosed Type 2 diabetes. Consequently, the pooled estimate may reflect detected rather than actual disease prevalence. The potential possibility of underdiagnosis may also be a concern in LMIC correctional facilities where screening infrastructure is limited. Third, publication bias is likely, as suggested by the marked asymmetry in the DOI plot and high LFK index of 3.58. While DOI plot asymmetry in prevalence meta‐analyses could reflect genuine heterogeneity rather than selective reporting, the magnitude observed here raises concern regarding the possibility of publication bias. Fourth, some included studies used convenience sampling, limiting representativeness. Several studies had relatively small sample sizes, and few provided detailed information on participant demographics. Additionally, important modifiers such as duration of incarceration, access to prison healthcare services, comorbid conditions, medication availability, nutritional patterns and physical activity were inconsistently explored, precluding more granular analyses. Fifth, data on diabetes subtypes, prediabetes and gestational diabetes were sparse. The lack of meta‐analysis of pooled estimates for these clinically important categories restricts understanding of the full metabolic disease spectrum within correctional facilities. Given that prediabetes represents an opportunity for early intervention, its underreporting is a missed preventive insight. Sixth, the review was limited to English‐language publications or studies with official English translations. While extensive regional database searching was performed, relevant non‐English studies could not have been captured or included, introducing language bias. Finally, because incarcerated populations differ demographically from general populations (e.g., younger age distribution, male predominance), direct comparisons with national prevalence estimates should be interpreted cautiously. Age‐standardized comparisons were not feasible due to insufficient reporting, and this could partially explain observed differences between correctional facility and community prevalence. Additionally, a low prevalence could mean higher mortality, possibly due to lack of proper treatment access, but this could not be further explored as the review's objectives were focused on prevalence rather than mortality. Some studies also exclude people with comorbidities that could influence the estimates. The review is also constrained by the inability to adjust for important clinical and behavioural confounders. Factors such as body mass index, diet quality within prisons, physical activity levels, substance use, HIV status, tuberculosis co‐infection and access to medical care are known to influence both the risk and detection of diabetes. However, these variables were inconsistently reported across studies and therefore could not be incorporated into subgroup or meta‐regression analyses in a meaningful way. This limits the ability to explore causal pathways or identify drivers of heterogeneity. Temporal heterogeneity also is a limitation. The included studies span multiple decades, during which both prison health systems and diagnostic practices have evolved substantially. Older studies may reflect historical conditions that are no longer representative of current prison healthcare infrastructure. Although meta‐regression did not identify publication year as a significant moderator, this may reflect limited statistical power rather than the absence of a true temporal effect. Subgroup analyses should not be interpreted as definitive, as some subgroups are based on a small number of studies or participants. Statistically significant differences between continents, countries, or other subgroups should not necessarily be interpreted as evidence of true epidemiological differences, as estimates may be influenced by the number and characteristics of contributing studies, differences in diabetes ascertainment and clinical and methodological heterogeneity. Country‐specific prevalence estimates in particular should be interpreted cautiously and may not be nationally representative, as some countries are represented by only one or a few studies conducted in individual correctional facilities. The limited number of studies contributing to some subgroups also reduces the power and reliability of these comparisons. This same consideration also applies to other subgroup analyses, including but not limited to those by continent and by correctional facility. As such, the findings of this review should be interpreted with great caution due to the inherent limitations of aggregating prevalence estimates across fundamentally different health system contexts.

### 4.3 Future Directions

Future primary and secondary research may benefit from exploring differences across healthcare systems in incarcerated institutions in LMICs with regards to diagnostic methods, geographical variability and contextual factors to potentially help explain the observed differences in diabetes prevalence. Future studies should also assess how diabetes ascertainment, participant characteristics and quality of prison healthcare influence reported prevalence. Additionally, future reviews can assess diabetes prevalence among incarcerated populations in high‐income countries. Future research may consider generating more comparable prevalence estimates through standardized diagnostic criteria, consistent reporting of prison characteristics and improved methodological quality.

## 5 Conclusion

This systematic review and meta‐analysis is the first to explore diabetes prevalence in incarcerated populations in the context of LMICs. As the pooled prevalence (3.7%) appears lower than global adult estimates, this difference should be interpreted cautiously given differences in population characteristics and the potential influence of variation in diabetes ascertainment, screening practices and other methodological limitations of the primary studies. Limited detection or underdiagnosis may be among the possible explanations for the observed difference, but this review did not directly assess undiagnosed diabetes. The very low certainty of evidence further emphasizes the need for higher‐quality, standardized epidemiologic research in this neglected population and this prevalence estimate should not be interpreted as definitive. As such, the prevalence estimate should be considered tentative and not definitive. From a clinical and public health perspective, correctional facilities may represent important settings for identifying and managing diabetes among incarcerated populations. Appropriate diabetes screening, access to essential medications, chronic disease management and continuity of care following release may be potentially relevant considerations for correctional health systems. However, the optimal approaches to screening and management in LMIC correctional settings require further evaluation as these were not evaluated within our review. Given the high heterogeneity and very low certainty of available evidence, these considerations should not be interpreted as evidence of intervention effectiveness. Future research may consider exploring stronger surveillance systems, standardized reporting and evaluation of feasible and context‐appropriate strategies for diabetes screening and management in incarcerated populations.

## Author Contributions

**Saad Arsalan Wasti:** conceptualization, methodology, software, validation, formal analysis, investigation, resources, data curation, writing – original draft, writing – review and editing, supervision, visualization, project administration. **Areesha Wasti:** conceptualization, software, methodology, formal analysis, validation, writing – review and editing. **Alisha Ahmed:** conceptualization, writing – original draft. **Beenish Sabir:** investigation, validation, methodology, data curation. **Ariba Asif:** investigation, data curation. **Sardar Noman Qayyum:** software, investigation, methodology, data curation. **Hammad Naveed:** investigation, methodology. **Noor ul Ain Saleem:** writing – original draft, writing – review and editing. **Muhammad Usman Iqbal:** writing – original draft, writing – review and editing. **Hamayle Saeed:** writing – original draft, writing – review and editing. **Dori Woldu:** validation, supervision, writing – original draft, writing – review and editing. **Shahad Abu Ahmad:** writing – original draft, writing – review and editing. **Muhammad Nabeel Saddique:** writing – review and editing, visualization, supervision.

## Funding

The authors have nothing to report.

## Consent

Consent for publication is not applicable as this study involves publicly available data.

## Conflicts of Interest

The authors declare no conflicts of interest.

## Supporting information


**Table S1:** PRISMA checklist.
**Table S2:** GATHER checklist.
**Table S3:** Database(s) with search strings and results.
**Table S4:** Other source(s) with search strings and results.
**Table S5:** Adapted‐GRADE framework.
**Table S6:** JBI appraisal [+ = Yes (1 Point); − = No (0 Point);? = Unclear (0.5 Point)].
**Figure S1:** Subgroup by correctional facility type.
**Figure S2:** Leave one out sensitivity analysis.
**Figure S3:** Baujat plot.
**Figure S4:** Bubble plot of meta‐regression with covariate year of publication.
**Figure S5:** Bubble plot of meta‐regression with covariate sample size.

## Data Availability

The data underlying this systematic review and meta‐analysis were obtained from publicly available sources. The data extraction form, extracted analytical dataset, and statistical code used for the analyses may be made available by the corresponding author upon reasonable request.
