# Supplementary material for: Prevalence of Diabetes in Low‐ and Middle‐Income Countries' Incarcerated Populations: A Systematic Review and Meta‐Analysis
Source: Endocrinol Diabetes Metab. 2026 Nov 1;9(6):e70374. doi: 10.1002/edm2.70374 (PMC13724223; doi:10.1002/edm2.70374)
Supplement: Supplementary file 1 [file EDM2-9-e70374-s001.docx]

**Supplementary File**

*Wasti et al., 2026*

**Supplementary Table S1.** PRISMA Checklist

| **Section and Topic** | **Item #** | **Checklist item** | **Location where item is reported** |
| --- | --- | --- | --- |
| **TITLE** | | |  |
| Title | 1 | Identify the report as a systematic review. | Title |
| **ABSTRACT** | | |  |
| Abstract | 2 | See the PRISMA 2020 for Abstracts checklist. | See Below |
| **INTRODUCTION** | | |  |
| Rationale | 3 | Describe the rationale for the review in the context of existing knowledge. | Page 5 |
| Objectives | 4 | Provide an explicit statement of the objective(s) or question(s) the review addresses. | Page 6 |
| **METHODS** | | |  |
| Eligibility criteria | 5 | Specify the inclusion and exclusion criteria for the review and how studies were grouped for the syntheses. | Page 7 |
| Information sources | 6 | Specify all databases, registers, websites, organisations, reference lists and other sources searched or consulted to identify studies. Specify the date when each source was last searched or consulted. | Page 7 |
| Search strategy | 7 | Present the full search strategies for all databases, registers and websites, including any filters and limits used. | Supplementary |
| Selection process | 8 | Specify the methods used to decide whether a study met the inclusion criteria of the review, including how many reviewers screened each record and each report retrieved, whether they worked independently, and if applicable, details of automation tools used in the process. | Page 7 |
| Data collection process | 9 | Specify the methods used to collect data from reports, including how many reviewers collected data from each report, whether they worked independently, any processes for obtaining or confirming data from study investigators, and if applicable, details of automation tools used in the process. | Page 8 |
| Data items | 10a | List and define all outcomes for which data were sought. Specify whether all results that were compatible with each outcome domain in each study were sought (e.g. for all measures, time points, analyses), and if not, the methods used to decide which results to collect. | Page 8 |
|  | 10b | List and define all other variables for which data were sought (e.g. participant and intervention characteristics, funding sources). Describe any assumptions made about any missing or unclear information. | Page 8 |
| Study risk of bias assessment | 11 | Specify the methods used to assess risk of bias in the included studies, including details of the tool(s) used, how many reviewers assessed each study and whether they worked independently, and if applicable, details of automation tools used in the process. | Page 8 |
| Effect measures | 12 | Specify for each outcome the effect measure(s) (e.g. risk ratio, mean difference) used in the synthesis or presentation of results. | Page 8 |
| Synthesis methods | 13a | Describe the processes used to decide which studies were eligible for each synthesis (e.g. tabulating the study intervention characteristics and comparing against the planned groups for each synthesis (item #5)). | Page 8 |
|  | 13b | Describe any methods required to prepare the data for presentation or synthesis, such as handling of missing summary statistics, or data conversions. | Page 9 |
|  | 13c | Describe any methods used to tabulate or visually display results of individual studies and syntheses. | Page 9 |
|  | 13d | Describe any methods used to synthesize results and provide a rationale for the choice(s). If meta-analysis was performed, describe the model(s), method(s) to identify the presence and extent of statistical heterogeneity, and software package(s) used. | Page 9 |
|  | 13e | Describe any methods used to explore possible causes of heterogeneity among study results (e.g. subgroup analysis, meta-regression). | Page 9 |
|  | 13f | Describe any sensitivity analyses conducted to assess robustness of the synthesized results. | Page 9 |
| Reporting bias assessment | 14 | Describe any methods used to assess risk of bias due to missing results in a synthesis (arising from reporting biases). | Supplementary |
| Certainty assessment | 15 | Describe any methods used to assess certainty (or confidence) in the body of evidence for an outcome. | Page 11 |
| **RESULTS** | | |  |
| Study selection | 16a | Describe the results of the search and selection process, from the number of records identified in the search to the number of studies included in the review, ideally using a flow diagram. | Figure 1 |
|  | 16b | Cite studies that might appear to meet the inclusion criteria, but which were excluded, and explain why they were excluded. | Figure 1 |
| Study characteristics | 17 | Cite each included study and present its characteristics. | Table 1 |
| Risk of bias in studies | 18 | Present assessments of risk of bias for each included study. | Supplementary |
| Results of individual studies | 19 | For all outcomes, present, for each study: (a) summary statistics for each group (where appropriate) and (b) an effect estimate and its precision (e.g. confidence/credible interval), ideally using structured tables or plots. | Figures |
| Results of syntheses | 20a | For each synthesis, briefly summarise the characteristics and risk of bias among contributing studies. | Figures and Tables |
|  | 20b | Present results of all statistical syntheses conducted. If meta-analysis was done, present for each the summary estimate and its precision (e.g. confidence/credible interval) and measures of statistical heterogeneity. If comparing groups, describe the direction of the effect. | Figures and Supplementary |
|  | 20c | Present results of all investigations of possible causes of heterogeneity among study results. | Figures and Supplementary |
|  | 20d | Present results of all sensitivity analyses conducted to assess the robustness of the synthesized results. | Supplementary |
| Reporting biases | 21 | Present assessments of risk of bias due to missing results (arising from reporting biases) for each synthesis assessed. | Supplementary |
| Certainty of evidence | 22 | Present assessments of certainty (or confidence) in the body of evidence for each outcome assessed. | SoF Table |
| **DISCUSSION** | | |  |
| Discussion | 23a | Provide a general interpretation of the results in the context of other evidence. | Page 15 |
|  | 23b | Discuss any limitations of the evidence included in the review. | Page 19 |
|  | 23c | Discuss any limitations of the review processes used. | Page 20 |
|  | 23d | Discuss implications of the results for practice, policy, and future research. | Pages 20 |
| **OTHER INFORMATION** | | |  |
| Registration and protocol | 24a | Provide registration information for the review, including register name and registration number, or state that the review was not registered. | Page 6 |
|  | 24b | Indicate where the review protocol can be accessed, or state that a protocol was not prepared. | Page 6 |
|  | 24c | Describe and explain any amendments to information provided at registration or in the protocol. | None |
| Support | 25 | Describe sources of financial or non-financial support for the review, and the role of the funders or sponsors in the review. | Title Page |
| Competing interests | 26 | Declare any competing interests of review authors. | Title Page |
| Availability of data, code and other materials | 27 | Report which of the following are publicly available and where they can be found: template data collection forms; data extracted from included studies; data used for all analyses; analytic code; any other materials used in the review. | These may be made available upon reasonable request to the authors |
| **PRISMA 2020 for Abstracts Checklist** | | | |
| **TITLE** |  |  | **Reported (Yes/No)** |
| Title | 1 | Identify the report as a systematic review. | Yes |
| **BACKGROUND** |  |  |  |
| Objectives | 2 | Provide an explicit statement of the main objective(s) or question(s) the review addresses. | Yes |
| **METHODS** |  |  |  |
| Eligibility criteria | 3 | Specify the inclusion and exclusion criteria for the review. | Yes |
| Information sources | 4 | Specify the information sources (e.g. databases, registers) used to identify studies and the date when each was last searched. | Yes |
| Risk of bias | 5 | Specify the methods used to assess risk of bias in the included studies. | Yes |
| Synthesis of results | 6 | Specify the methods used to present and synthesise results. | Yes |
| **RESULTS** |  |  |  |
| Included studies | 7 | Give the total number of included studies and participants and summarise relevant characteristics of studies. | Yes |
| Synthesis of results | 8 | Present results for main outcomes, preferably indicating the number of included studies and participants for each. If meta-analysis was done, report the summary estimate and confidence/credible interval. If comparing groups, indicate the direction of the effect (i.e. which group is favoured). | Yes |
| **DISCUSSION** |  |  |  |
| Limitations of evidence | 9 | Provide a brief summary of the limitations of the evidence included in the review (e.g. study risk of bias, inconsistency and imprecision). | Yes |
| Interpretation | 10 | Provide a general interpretation of the results and important implications. | Yes |
| **OTHER** |  |  |  |
| Funding | 11 | Specify the primary source of funding for the review. | Yes |
| Registration | 12 | Provide the register name and registration number. | Yes |

**Supplementary Table S2:** GATHER Checklist

| **Item #** | **Section** | **Checklist item** | **Reported on page #** |
| --- | --- | --- | --- |
| 1 | Objectives and funding | Define the indicator(s), populations (including age, sex, and geographic entities), and time period(s) for which estimates were made. | Page 5, Table 1 |
| 2 | Objectives and funding | List the funding sources for the work. | Title Page |
| 3 | Data inputs (synthesized data) | Describe how the data were identified and how the data were accessed. | Page 6 |
| 4 | Data inputs (synthesized data) | Specify the inclusion and exclusion criteria. Identify all ad-hoc exclusions. | Page 6 |
| 5 | Data inputs (synthesized data) | Provide information on all included data sources and their main characteristics. For each data source used, report reference information or contact name/institution, population represented, data collection method, year(s) of data collection, sex and age range, diagnostic criteria or measurement method, and sample size, as relevant. | Page 6 |
| 6 | Data inputs (synthesized data) | Identify and describe any categories of input data that have potentially important biases (e.g., based on characteristics listed in item 5). | Supplementary |
| 7 | Data inputs (non-synthesized data) | Describe and give sources for any other data inputs that contribute to the analysis but were not synthesized as part of the study. | Table 1 |
| 8 | Data inputs (all data) | Provide all data inputs in a file format from which data can be efficiently extracted (e.g., a spreadsheet rather than a PDF), including all relevant metadata listed in item 5. For data that cannot be shared due to ethical or legal reasons, provide a contact name or institution that retains the rights to the data. | Forest Plots |
| 9 | Data analysis | Provide a conceptual overview of the data analysis method. A diagram may be helpful. | Page 10 |
| 10 | Data analysis | Provide a detailed description of all steps of the analysis, including mathematical formulae. Cover data cleaning, preprocessing, adjustments, weighting of data sources, and mathematical or statistical models, as relevant. | Page 10 |
| 11 | Data analysis | Describe how candidate models were evaluated and how the final model(s) were selected. | Page 10 |
| 12 | Data analysis | Provide results of any evaluation of model performance, if done, and results of relevant sensitivity analyses. | Page 10 |
| 13 | Data analysis | Describe methods for calculating uncertainty of the estimates. State which sources of uncertainty were and were not accounted for. | Page 11 |
| 14 | Data analysis | State how analytic or statistical source code used to generate estimates can be accessed. | Data Availability Statement |
| 15 | Results and discussion | Provide published estimates in a file format from which data can be efficiently extracted. | Forest Plots |
| 16 | Results and discussion | Report a quantitative measure of the uncertainty of the estimates (e.g., uncertainty intervals). | Forest Plots |
| 17 | Results and discussion | Interpret results in light of existing evidence. If updating previous estimates, describe reasons for changes. | Discussion |
| 18 | Results and discussion | Discuss limitations of the estimates, including modeling assumptions or data limitations affecting interpretation. | Discussion |

**Supplementary Table S3.** Database(s) with Search Strings and Results.

| **Database(s)** | **Search String** | **Results** |
| --- | --- | --- |
| ***PubMed*** | #1  ((((((((((((((((((((((((((((((((((((((("Diabetes Mellitus"[Mesh]) OR ("Diabetes, Gestational"[Mesh])) OR ("Diabetes Mellitus, Type 1"[Mesh])) OR ("Diabetes Mellitus, Type 2"[Mesh])) OR (Diabet*[Title/Abstract])) OR ("Diabetes mellitus"[Title/Abstract])) OR (DM[Title/Abstract])) OR (T1DM[Title/Abstract])) OR (IDDM[Title/Abstract])) OR ("T1-DM"[Title/Abstract])) OR ("Type 1 Diabet*"[Title/Abstract])) OR ("Type I Diabet*"[Title/Abstract])) OR ("Type I DM"[Title/Abstract])) OR ("Type 1 DM"[Title/Abstract])) OR ("Insulin-Dependent Diabet*"[Title/Abstract])) OR ("Juvenile-Onset Diabet*"[Title/Abstract])) OR ("Autoimmune Diabet*"[Title/Abstract])) OR ("Sudden-Onset Diabet*"[Title/Abstract])) OR ("Ketosis-Prone Diabet*"[Title/Abstract])) OR ("Brittle Diabet*"[Title/Abstract])) OR (GDM[Title/Abstract])) OR ("Gestational Diabet*"[Title/Abstract])) OR ("Pregnancy-Induced Diabet*"[Title/Abstract])) OR (NIDDM[Title/Abstract])) OR (T2DM[Title/Abstract])) OR ("T2-DM"[Title/Abstract])) OR ("Type 2 DM"[Title/Abstract])) OR ("Type II DM"[Title/Abstract])) OR ("Type 2 Diabet*"[Title/Abstract])) OR ("Type II Diabet*"[Title/Abstract])) OR ("Stable Diabet*"[Title/Abstract])) OR ("Adult-Onset Diabet*"[Title/Abstract])) OR ("Slow-Onset Diabet*"[Title/Abstract])) OR ("Ketosis-Resistant Diabet*"[Title/Abstract])) OR ("Noninsulin Dependent Diabet*"[Title/Abstract])) OR ("Non-Insulin Dependent Diabet*"[Title/Abstract])) OR ("Maturity-Onset Diabet*"[Title/Abstract])) OR (MODY[Title/Abstract])) OR (Prediabet*[Title/Abstract])) OR ("Pre-diabet*"[Title/Abstract])  #2  ((((((((((((((((((("Prisons"[Mesh]) OR ("Correctional Facilities"[Mesh])) OR ("Prisoners"[Mesh])) OR (Prison*[Title/Abstract])) OR (Penitentia*[Title/Abstract])) OR ("Correctional Facili*"[Title/Abstract])) OR ("Correction Facili*"[Title/Abstract])) OR ("Penal Institu*"[Title/Abstract])) OR ("Penal Facili*"[Title/Abstract])) OR (Gaol*[Title/Abstract])) OR (Jail*[Title/Abstract])) OR ("Correctional Institu*"[Title/Abstract])) OR ("Correction Institu*"[Title/Abstract])) OR ("Detention camp*"[Title/Abstract])) OR ("Internment camp*"[Title/Abstract])) OR ("Incarcerated Individual*"[Title/Abstract])) OR ("Imprisoned Individual*"[Title/Abstract])) OR (Inmate*[Title/Abstract])) OR ("Detained Person*"[Title/Abstract])) OR (Detainee*[Title/Abstract])  #3  #1 AND #2 | **300** |
| ***Embase*** | ('diabetes mellitus'/exp OR 'diabetes' OR 'diabetes mellitus' OR 'diabetic' OR 'diabets' OR 'unspecified diabetes mellitus') AND ('correctional facility'/exp OR 'correctional facilities' OR 'correctional facility' OR 'correctional institution' OR 'gaol' OR 'jail' OR 'jails' OR 'penal facilities' OR 'penal institution' OR 'penitentiary' OR 'prison' OR 'prisons' OR 'prisoner'/exp OR 'incarcerated offender' OR 'incarcerated offenders' OR 'incarcerated population' OR 'inmate' OR 'inmates' OR 'prisoner' OR 'prisoners') | **777** |
| ***Scopus*** | #1  TITLE-ABS-KEY(Diabet*) OR TITLE-ABS-KEY("Diabetes mellitus") OR TITLE-ABS-KEY(DM) OR TITLE-ABS-KEY(T1DM) OR TITLE-ABS-KEY(IDDM) OR TITLE-ABS-KEY("T1-DM") OR TITLE-ABS-KEY("Type 1 Diabet*") OR TITLE-ABS-KEY("Type I Diabet*") OR TITLE-ABS-KEY("Type I DM") OR TITLE-ABS-KEY("Type 1 DM") OR TITLE-ABS-KEY("Insulin-Dependent Diabet*") OR TITLE-ABS-KEY("Juvenile-Onset Diabet*") OR TITLE-ABS-KEY("Autoimmune Diabet*") OR TITLE-ABS-KEY("Sudden-Onset Diabet*") OR TITLE-ABS-KEY("Ketosis-Prone Diabet*") OR TITLE-ABS-KEY("Brittle Diabet*") OR TITLE-ABS-KEY(GDM) OR TITLE-ABS-KEY("Gestational Diabet*") OR TITLE-ABS-KEY("Pregnancy-Induced Diabet*") OR TITLE-ABS-KEY(NIDDM) OR TITLE-ABS-KEY(T2DM) OR TITLE-ABS-KEY("T2-DM") OR TITLE-ABS-KEY("Type 2 DM") OR TITLE-ABS-KEY("Type II DM") OR TITLE-ABS-KEY("Type 2 Diabet*") OR TITLE-ABS-KEY("Type II Diabet*") OR TITLE-ABS-KEY("Stable Diabet*") OR TITLE-ABS-KEY("Adult-Onset Diabet*") OR TITLE-ABS-KEY("Slow-Onset Diabet*") OR TITLE-ABS-KEY("Ketosis-Resistant Diabet*") OR TITLE-ABS-KEY("Noninsulin Dependent Diabet*") OR TITLE-ABS-KEY("Non-Insulin Dependent Diabet*") OR TITLE-ABS-KEY("Maturity-Onset Diabet*") OR TITLE-ABS-KEY(MODY) OR TITLE-ABS-KEY(Prediabet*) OR TITLE-ABS-KEY("Pre-diabet*")  #2  TITLE-ABS-KEY(Prison*) OR TITLE-ABS-KEY(Penitentia*) OR TITLE-ABS-KEY("Correctional Facili*") OR TITLE-ABS-KEY("Correction Facili*") OR TITLE-ABS-KEY("Penal Institu*") OR TITLE-ABS-KEY("Penal Facili*") OR TITLE-ABS-KEY(Gaol*) OR TITLE-ABS-KEY(Jail*) OR TITLE-ABS-KEY("Correctional Institu*") OR TITLE-ABS-KEY("Correction Institu*") OR TITLE-ABS-KEY("Detention camp*") OR TITLE-ABS-KEY("Internment camp*") OR TITLE-ABS-KEY("Incarcerated Individual*") OR TITLE-ABS-KEY("Imprisoned Individual*") OR TITLE-ABS-KEY(Inmate*) OR TITLE-ABS-KEY("Detained Person*") OR TITLE-ABS-KEY(Detainee*)  #3  #1 AND #2 | **610** |
| ***Web of Science*** | #1  TS=(Diabet* OR "Diabetes mellitus" OR DM OR T1DM OR IDDM OR "T1-DM" OR "Type 1 Diabet*" OR "Type I Diabet*" OR "Type I DM" OR "Type 1 DM" OR "Insulin-Dependent Diabet*" OR "Juvenile-Onset Diabet*" OR "Autoimmune Diabet*" OR "Sudden-Onset Diabet*" OR "Ketosis-Prone Diabet*" OR "Brittle Diabet*" OR GDM OR "Gestational Diabet*" OR "Pregnancy-Induced Diabet*" OR NIDDM OR T2DM OR "T2-DM" OR "Type 2 DM" OR "Type II DM" OR "Type 2 Diabet*" OR "Type II Diabet*" OR "Stable Diabet*" OR "Adult-Onset Diabet*" OR "Slow-Onset Diabet*" OR "Ketosis-Resistant Diabet*" OR "Noninsulin Dependent Diabet*" OR "Non-Insulin Dependent Diabet*" OR "Maturity-Onset Diabet*" OR MODY OR Prediabet* OR "Pre-diabet*")  #2  TS=(Prison* OR Penitentia* OR "Correctional Facili*" OR "Correction Facili*" OR "Penal Institu*" OR "Penal Facili*" OR Gaol* OR Jail* OR "Correctional Institu*" OR "Correction Institu*" OR "Detention camp*" OR "Internment camp*" OR "Incarcerated Individual*" OR "Imprisoned Individual*" OR Inmate* OR "Detained Person*" OR Detainee*)  #3  #1 AND #2 | **287** |

**Supplementary Table S4.** Other Source(s) with Search Strings and Results.

| **Other Sources** | **Search String** | **Results** |
| --- | --- | --- |
| ***Websites**** | Diabetes AND (Prison OR prisoner OR prisons OR prisoners OR incarcerated OR jail OR felon OR felons)  *DOAJ.org; sagepub.com; scholar.google.com; springer.com; sciencedirect.com; onlinelibrary.wiley.com; researchgate.net; academia.edu; oversea.cnki.net; scielo.br; LILACS; AJOL; PakMediNet; WPRIM; SID; Global Index Medicus | **23** |
| ***Citation Chaining*** | Done Through Google Scholar and Reference Lists | **45** |

**Supplementary Table S5:** Adapted-GRADE Framework

| **Domain influencing certainty of evidence** | **How the domain was applied in this review** |
| --- | --- |
| **Risk of bias (study limitations)** | As prevalence studies are typically observational, cross-sectional or cohort designs conducted in representative prison populations were considered appropriate starting designs. Risk of bias was evaluated using the Joanna Briggs Institute (JBI) critical appraisal tool for prevalence studies. Evidence was downgraded when important methodological limitations were identified. |
| **Imprecision** | Precision was assessed by examining the width of confidence intervals around individual and pooled prevalence estimates. Wide confidence intervals, reflecting small sample sizes or sparse outcome data, or widely varying estimates, or very few studies, were considered indicative of imprecision and resulted in downgrading of certainty. |
| **Inconsistency** | Inconsistency was evaluated by adapting data from the revised GRADE guidelines found in the GRADE book (book.gradepro.org/guideline/inconsistency). Statistical heterogeneity does not provide a meaningful indication of inter-study variably, as I^2 values *are* expected to be high in meta-analyses of prevalence. Greater emphasis was placed on prediction intervals and the clinical and epidemiological spread of estimates rather than I^2 alone. According to current GRADE guidance, downgrades were only to be considered if heterogeneity could not be explained by inter-study variations in the PICO elements e.g. different countries, different study designs, different participant demographics. Since heterogeneity was attributable to these factors, a downgrade was not considered. |
| **Indirectness** | Indirectness was assessed based on how closely the included studies aligned with the review question, which was to evaluate the prevalence of diabetes among the incarcerated population in LMICs. Indirectness was not a concern in the review. |
| **Publication bias** | The likelihood of publication or selective reporting bias was assessed using DOI plots and the Luis Furuya-Kanamori (LFK) index. Asymmetry suggestive of missing studies or selective reporting resulted in downgrading the certainty of evidence. |
| **Upgrading factors (e.g., large effects)** | Currently, no validated criteria exist for upgrading certainty based on large or dose-response effects in prevalence studies. Therefore, no upgrading of certainty ratings was applied in this review. |

**Supplementary Table S6:** JBI Appraisal [+ = Yes (1 Point); - = No (0 Point); ? = Unclear (0.5 Point)]

| **Serial Number** | **Author** | **D1** | **D2** | **D3** | **D4** | **D5** | **D6** | **D7** | **D8** | **D9** | **Overall** |
| --- | --- | --- | --- | --- | --- | --- | --- | --- | --- | --- | --- |
| **1** | Bolivar | + | + | + | + | + | + | + | + | ? | 8.5/9 |
| **2** | Monteiro | + | + | + | + | + | - | - | + | + | 7/9 |
| **3** | Njonnou | + | + | + | + | + | + | + | + | + | 9/9 |
| **4** | Serra | + | + | + | + | + | + | + | + | + | 9/9 |
| **5** | Leal | + | + | - | + | + | + | + | + | ? | 7.5/9 |
| **6** | Ahmed | + | + | + | + | ? | ? | ? | - | ? | 6/9 |
| **7** | Abukari | ? | - | + | + | + | + | + | + | ? | 7/9 |
| **8** | Lai | ? | + | + | + | + | + | + | + | + | 8.5/9 |
| **9** | Narayan | ? | + | - | + | + | ? | + | + | + | 7/9 |
| **10** | Bautista-Arredondo | + | + | + | + | - | + | - | + | + | 7/9 |
| **11** | Demissie | + | + | + | + | + | + | + | + | + | 9/9 |
| **12** | Galvão | ? | - | - | + | ? | - | ? | - | + | 3.5/9 |
| **13** | Qadir | + | - | + | + | ? | - | - | - | ? | 4/9 |
| **14** | Rao | + | + | + | + | + | - | - | + | + | 6/9 |
| **15** | Sabir | + | + | + | + | + | + | + | + | + | 9/9 |
| **16** | Kumar | + | + | + | + | + | ? | ? | + | + | 8/9 |
| **17** | Wickramatilake | + | + | + | + | + | - | - | + | + | 7/9 |
| **18** | Bellad | + | + | + | + | + | ? | ? | + | + | 8/9 |
| **19** | Shah | + | + | + | + | + | - | - | + | + | 7/9 |
| **20** | Jovanovska | + | + | + | + | + | - | - | + | + | 7/9 |
| **21** | Morasert | + | + | + | + | + | - | - | + | + | 7/9 |
| **22** | Saeed | + | + | - | + | ? | - | - | - | + | 4.5/9 |
| **23** | Rueda | + | + | + | + | ? | - | - | - | + | 5.5/9 |

**Supplementary Figure S1.** Subgroup by correctional facility type

**Supplementary Figure S2.** Leave one out sensitivity analysis

**Supplementary Figure S3.** Baujat Plot

**Supplementary Figure S4.** Bubble Plot of Meta-Regression with Covariate Year of Publication

**Supplementary Figure S5.** Bubble Plot of Meta-Regression with Covariate Sample Size
